# Low-Osmolality Carbohydrate–Electrolyte Solution Ingestion Avoid Fluid Loss and Oxidative Stress after Exhaustive Endurance Exercise

**DOI:** 10.3390/antiox9040336

**Published:** 2020-04-20

**Authors:** Wen-Ching Huang, Yu-Tang Tung, Mai-Szu Wu, Ming-Che Liu, Tsai-Jung Lin, Ming-Ta Yang

**Affiliations:** 1 Department of Exercise and Health Science, National Taipei University of Nursing and Health Sciences, Taipei 11219, Taiwan; wenching@ntunhs.edu.tw; 2 Graduate Institute of Metabolism and Obesity Sciences, Taipei Medical University, Taipei 11301, Taiwan; f91625059@tmu.edu.tw; 3 TMU Research Center of Urology and Kidney, Taipei Medicine University, Taipei 110301, Taiwan; maiszuwu@gmail.com; 4 Division of Nephrology, Department of Internal Medicine, School of Medicine, College of Medicine, Taipei Medical University, Taipei City 110301, Taiwan; 5 Division of Nephrology, Department of Internal Medicine, Taipei Medical University-Shuang Ho Hospital, New Taipei City 23561, Taiwan; 6 Department of Urology, Taipei Medical University Hospital, Taipei 110301, Taiwan; d204097002@tmu.edu.tw; 7 Clinical Research Center, Taipei Medical University Hospital, Taipei 110301, Taiwan; 8 School of Dental Technology, College of oral Medicine, Taipei Medical University, Taipei 110301, Taiwan; 9 Graduate Institute of Clinical Medicine, College of Medicine, Taipei Medical University, Taipei 110301, Taiwan; 10 School of Medicine, College of Medicine, Taipei Medical University, Taipei 110301, Taiwan; b101104113@tmu.edu.tw; 11 Center for General Education, Taipei Medical University, Taipei 110301, Taiwan

**Keywords:** rehydration solution, oxidative stress, antioxidation, exhaustive endurance exercise, dehydration

## Abstract

Low-osmolality carbohydrate–electrolyte solution (LCS) ingestion can replace losses from exercise-induced dehydration, but the benefits of LCS ingestion strategy after exhaustive endurance exercise (EEE) remain unknown. The present study evaluated the effects of LCS ingestion on dehydration, oxidative stress, renal function, and aerobic capacity after EEE. In our study with its double-blind, crossover, counterbalanced design, 12 healthy male participants were asked to consume LCS (150 mL four times per hour) or placebo (water) 1 h before and 1 h after EEE. All participants completed a graded exercise test to exhaustion on a treadmill for the determination of maximal oxygen consumption (${\dot{V}O}_{2\max}$), applied to further intensity calibration, and then completed the EEE test. The average heart rate, maximal heart rate, running time to exhaustion, and peak oxygen uptake (VO_2peak_) were recorded during the exercise period. The participants’ body weight was recorded at different time points before and after the EEE to calculate the dehydration rate. Blood samples were drawn at baseline and before, immediately after, 1 h after, and 2 h after EEE to determine indicators of oxidative stress and renal function. The results indicated that the dehydration rates in participants with LCS ingestion at 15 min, 30 min, and 45 min after EEE were significantly lower than in participants with placebo ingestion (−1.86 ± 0.47% vs. −2.24 ± 0.72%; −1.78 ± 0.50% vs. −2.13 ± 0.74%; −1.54 ± 0.51% vs. −1.94 ± 0.72%, respectively; *p* < 0.05). In addition, the concentration of catalase in participants with LCS ingestion immediately after EEE was significantly higher than in participants with placebo ingestion (2046.21 ± 381.98 nmol/min/mL vs. 1820.37 ± 417.35 nmol/min/mL; *p* < 0.05). Moreover, the concentration of protein carbonyl in participants with LCS ingestion immediately after EEE was slightly lower than in participants with placebo ingestion (2.72 ± 0.31 nmol carbonyl/mg protein vs. 2.89 ± 0.43 nmol carbonyl/mg protein; *p* = 0.06). No differences were noted for other variables. Our findings conclude that LCS ingestion can effectively avoid fluid loss and oxidative stress after EEE. However, LCS ingestion had no benefits for renal function or aerobic capacity.

## 1. Introduction

Water, the dominant ingredient in the human body, is essential to maintain physiological activities and functions for metabolism, enzymatic reaction, and thermoregulation. The water balance in the body’s fluids can be considered the sum of acquisition (dietary and metabolic) and loss (through respiration, urine, and sweat), and a 2% fluid deficit of body weight (BW) can be deleterious to cognitive function and aerobic exercise performance, particularly in hot weather [1,2]. Dehydration syndrome can be observed clinically as a result of infectious acute diarrhea, and the use of oral rehydration solution (ORS) is strongly recommended as a therapeutic strategy [3]. The World Health Organization (WHO) announced that ORS containing glucose (13.5 g/L), sodium (75 mEq/L), chloride (65 mEq/L), potassium (20 mEq/L), and citrate (30 mEq/L) with osmotic pressure of 245 mOsm/L could effectively replace fluid lost due to diarrhea to decrease morbidity and mortality. The original formula of ORS was developed in 1975 by the WHO, and it was later modified with different ingredient types and lower osmotic pressure for greater rehydration efficiency [4]. Physical activity, especially high-intensity endurance exercise and regular training, is another activity that results in the loss of body fluid and attainment of hypohydration status. Excessive sweat loss (water and electrolytes, especially sodium) after prolonged exercise could cause hypovolemia, and excessive hypotonic fluid consumption (water or sports drinks) could result in hyponatremia [5].

Dehydration often occurs during prolonged exercise; it may impair aerobic exercise performance [6]. Athletes with hypohydration have to rehydrate as soon as possible for upcoming competitions in order to optimize their physiological condition; pre-exercise hypohydration could impede their ${\dot{V}O}_{2\mathrm{peak}}$, ${\dot{V}O}_{2}$ at lactate threshold (${\dot{V}O}_{2\mathrm{LT}}$), and aerobic performance [7]. The thirst sensation could be considered as a perceived index of when athletes should consume fluids at their discretion during exercise [8]. However, the thirst sensation may not directly represent physiological conditions of euhydration and hypohydration, and an individualized rehydration plan and fluid type consumed could be important issues for euhydration after endurance exercise [9]. Hypohydration could also induce oxidative stress and impair cognitive function, possibly through an increase of osmolality, vasopressin, and critical mechanisms regulating cerebral circulation [10]. The free radicals generated by the oxidative respiration reaction are balanced by endogenous antioxidant system homeostasis [11], and endurance exercise could significantly induce oxidation, inflammatory biomarkers, and redox-related genes, including COX-2 and Nrf2 [12]. The amelioration of oxidative stress and inflammation could also improve exercise performance and lead to a healthier condition according to a nutritional supplementation and training model [13,14].

In recent years, the number of people who engage in cycling and running has rapidly increased, for the purpose of not only health promotion but also self-fulfillment. Most runners and cyclists have experienced performance decrements because of dehydration, and surveillance of hydration levels or specific hydration strategies are warranted for safety and health reasons [15]. Physical injury and physiological impacts are important factors in endurance exercise. With regard to physiological impacts, the prevalence of exercise-associated hyponatremia depends on exercise duration, the type of sports activity engaged in, the participant’s sex, and ambient temperature during exercise [16]. The fluid replacement guidelines of the American College of Sports Medicine (ACSM) might lead people to believe that overdrinking is encouraged, and it might increase the risk of hyponatremia among marathoners [17]. However, in its recent report [2], the ACSM recommended that athletes should achieve euhydration prior to exercise by consuming a fluid volume equivalent to 5–10 mL/kg BW in the 2 to 4 h before exercise to allow sufficient time for excess fluid to be voided. Thus, appropriate rehydration strategies and ingredient formulations taken over different durations still require further investigation for better physiological adaptation and health maintenance [18].

In the present study, we reformulated and modified an ORS to elucidate euhydration efficiency and possible supplementation strategies. In addition, physiological indexes, including oxidant stress and renal function, were assessed to determine the effects of low-osmolality carbohydrate–electrolyte solution (LCS) ingestion. The results will allow amateur athletes to make better-informed decisions about their endurance training with regard to euhydration.

## 2. Materials and Methods

### 2.1. Participants

Twelve healthy male participants were recruited from Taipei Medical University. Individuals with cardiovascular, liver, renal, diabetes, and autoimmune diseases were excluded. The participants were requested not to consume other nutritional supplements, to avoid alcohol drinking, and to maintain their regular live styles during the study. All participants provided written informed consent to participate in the study. The anthropometric data of the participants were as follows: Average age 22.42 ± 1.08 years, average weight 70.00 ± 7.03 kg, and average height 175.67 ± 5.63 cm. Approval for the study was obtained from the Human Research Ethics Committee of the Taipei Medical University, Taipei, Taiwan (no. N201902017).

### 2.2. LCS

The ingredients of the LCS (Sport Hydro^TM^; Panion & BF Biotech, Taipei, Taiwan) in the present study were modified from the ORS of the WHO-announced composition [4], which contains electrolytes (sodium (75 mEq/L), chloride (65 mEq/L), potassium (20 mEq/L)) and carbohydrates (glucose and maltose (75 mmol/L)) with osmolality of 245 mOsm/L. The LCS was reconstituted with water 20 min before ingestion at the indicated time points, and water was used as a placebo.

### 2.3. Experimental Design

**Figure 1:**
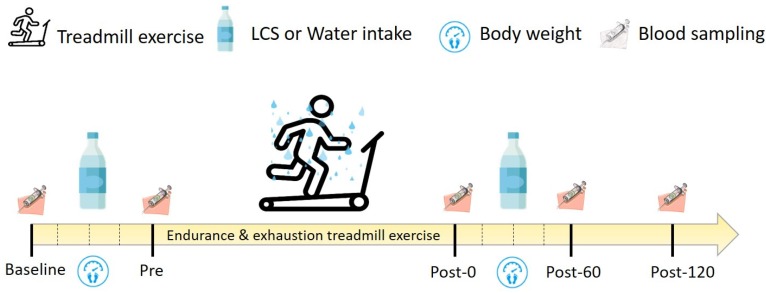
Experimental scheme. The experiment was based on a crossover double-blind design. The participants engaged in the treadmill exercise for 60 min with 70% maximal oxygen consumption (${\dot{V}O}_{2\max}$) intensity, followed by 90% ${\dot{V}O}_{2\max}$ until exhaustion. The indicated fluid was ingested at a rate of 600 mL/h (150 mL/15 min) before and after exhaustive endurance exercise. The participants’ BW information and blood samples were collected at indicated time points. LCS, low-osmolality carbohydrate–electrolyte solution; Pre, Pre-exercise; Post-0, Immediately after exercise; Post-60, 60 min after exercise; Post-120, 120 min after exercise.

A double-blind, counterbalanced, crossover experimental design was adopted to investigate the effects of LCS consumption on exhaustive endurance exercise (EEE). Washout periods of at least 3 weeks were used, and the participants also were asked to avoid engaging in any strenuous exercise, staying up late, smoking, and consuming nutritional supplements and alcoholic beverages. Maximal oxygen consumption (${\dot{V}O}_{2\max}$), was measured 1 week before the official experiment and adjusted for the intensity of 70% ${\dot{V}O}_{2\max}$ [19] for 1-h endurance treadmill exercise, followed by 90% ${\dot{V}O}_{2\max}$ to exhaustion. The participants consumed the standard breakfast (including 130 g of bread and 450 mL of rice and peanut milk with an energy content approximately 648 kcal) 1 h before baseline, and blood samples were collected at baseline, before exercise (Pre), immediately after exercise (Post-0), 60 min after exercise (Post-60), and 120 min after exercise (Post-120). The LCS was ingested 1 h before and 1 h after EEE in a quantity of 600 mL (150 mL/15 min) for hydration. In addition, the participants’ BW was recorded at baseline, 15 min after exercise (Post-15), 30 min after exercise (Post-30), 45 min after exercise (Post-45), and 60 min after exercise (Post-60) to calculate dehydration rates. Blood samples were further analyzed for related oxidative stress markers, electrolytes, osmolality, renal function, and inflammatory response. Also, urine was sampled 1 h before and 1 h after EEE for electrolytes and osmolality. The detailed procedures are detailed in Figure 1.

### 2.4. Maximal Oxygen Consumption Assessment and Exercise Intensity

The complete protocol was conducted according to a previous study to evaluate the participants’ ${\dot{V}O}_{2\max}$ aerobic capacity through a continuously graded exercise test [19]. The participants warmed up for 3 min to achieve a heart rate (HR) of less than 150 beats/min. After warmup, the treadmill (pulsar; h/p/cosmos, Nussdorf, Germany) speed was begun at 2.0 m/s and increased by 0.5 m/s every 4 min until the participant reached exhaustion. Then, the intensity was further elevated in increments of 0.5 m/s every 2 min until the participant reached exhaustion. Expired gas, ${\dot{V}O}_{2}$, and ${\dot{V}\mathrm{CO}}_{2}$ were analyzed by gas analysis (Vmax Spectra 29c; SensorMedics, Yorba Linda, CA), and HR was monitored at the same time. Individual ${\dot{V}O}_{2\max}$ was identified according to the following criteria: (1) Rating of perceived exertion greater than 18, (2) HR within 15 beats/min of individual predicted HRmax, and (3) respiratory exchange ratio greater than 1.1.

### 2.5. Biochemical Variables

In the present study, blood samples were collected from the participants at baseline and at the Pre, Post-0, Post-60, and Post-120 time points (Figure 1). The samples were collected into tubes with and without anticoagulant and then centrifuged at 3000× *g* for 10 min for plasma and serum isolation. The plasma and serum were stored at −20 °C for further analysis. The creatinine, C-reactive protein (CRP), sodium, potassium, chloride, and osmolality in plasma were assessed using an automatic biochemical analyzer (Siemens, Erlangen, Germany), and electrolytes in urine were also analyzed (AU5800; Beckman Coulter, Brea, CA, USA). Both plasma and serum were subjected to osmotic analysis (Osmomat 030; Gonotec, Berlin, Germany) for osmolality. In addition, creatinine and age were calculated to estimate glomerular filtration rate (eGFR), and this equation is follows: 186 × (creatinine)^−1.154^ × (age)^−0.203^ [20].

### 2.6. Oxidative Stress Evaluation

The indexes regarding oxidation (thiobarbituric acid reactive substances (TBARS), protein carbonyl (PC)), and antioxidation (glutathione peroxidase (GPx), superoxide dismutase (SOD), catalase) were measured using colorimetric kits for the LCS effects on exercise-induced redox homeostasis. The procedures were conducted according to the instructions for the kits, and the results were assessed using an enzyme-linked immunosorbent assay (PowerWave XS2; BioTek, Winooski, VT, USA).

### 2.7. Statistics

Data are expressed as mean ± standard deviation. One-way repeated-measures analysis of variance was performed to examine the differences between different time points, and a paired *t* test was used to examine the differences between different treatments. Analyses were performed using IBM SPSS version 22.0 software (IBM, Armonk, NY, USA). A *p* value < 05 was considered statistically significant.

## 3. Results

### 3.1. Effects of LCS on EEE-Induced Dehydration

**Figure 2:**
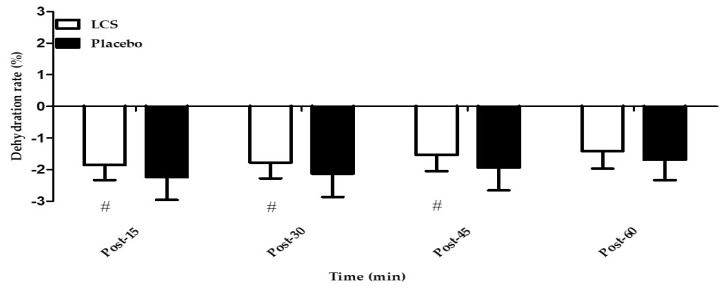
Changes in dehydration rate after exhaustive endurance exercise. The dehydration rate was calculated using difference ratios compared with baseline body weight at the indicated time points (45, 30, and 15 min) after exhaustive endurance exercise. #Significant (*p* < 0.05) difference between conditions. LCS, low-osmolality carbohydrate–electrolyte solution; Post-15, 15 min after exercise; Post-30, 30 min after exercise; Post-45, 45 min after exercise; Post-60, 60 min after exercise.

The participants’ BW was measured at baseline and at indicated time points after EEE (Post-15, Post-30, and Post-45) for the evaluation of dehydration. Fluid (LCS or water) was ingested at those time points. As shown in Figure 2, the decreasing percentage of BW in the LCS ingestion was significantly lower than that in the placebo ingestion after EEE at the Post-15, Post-30, and Post-45 time points (−1.86 ± 0.47% vs. −2.24 ± 0.72%; −1.78 ± 0.50% vs. −2.13 ± 0.74%; −1.54 ± 0.51% vs. −1.94 ± 0.72%, respectively; *p* < 0.05, statistical power = 0.6).

### 3.2. Effects of LCS on Renal Function During EEE

**Figure 3:**
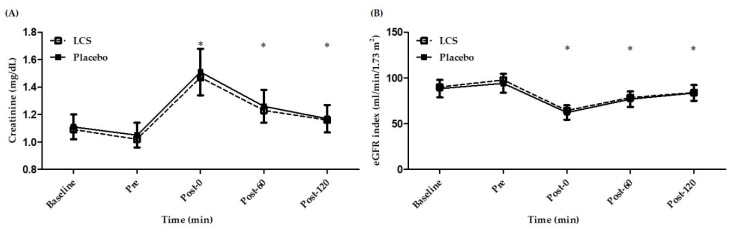
Changes in (**A**) creatinine and (**B**) estimated glomerular filtration rate (eGFR) after exhaustive endurance exercise. * Significant (*p* < 0.05) difference from baseline. LCS, low-osmolality carbohydrate–electrolyte solution; Pre, Pre-exercise; Post-0, Immediately after exercise; Post-60, 60 min after exercise; Post-120, 120 min after exercise.

Renal function could be evaluated through the direct measurement of creatinine and further assessed by sex and age to provide an estimated glomerular filtration rate (eGFR). As shown in Figure 3A, plasma creatinine levels were significantly increased after EEE and gradually recovered to normal baseline levels. As shown in Figure 3B, eGFR was significantly decreased after exercise (Post-0, Post-60, and Post-120) but then gradually returned to baseline level. However, no significant differences were observed between the two treatments.

### 3.3. Effects of LCS on Electrolytes and Osmolality

No significant differences between the LCS and placebo conditions were observed for electrolytes and osmolality (Table 1). LCS consumption significantly elevated electrolytes (sodium, chloride, and potassium) and decreased osmolality from euhydration at the pre-exercise time point. The equivalent placebo ingestion significantly increased osmolality but not electrolytes. After EEE, electrolytes and osmolality significantly increased from the baseline and Pre time points. Fluid ingestion after exercise achieved significant increases in rehydration through improvements in osmolality, and electrolyte concentrations (sodium and chloride) also significantly decreased to reflect rehydration in both conditions. Electrolyte levels and osmolality returned to baseline levels at Post-120, but sodium and potassium levels in the LCS group were still significantly higher than at baseline. In urine samples (Table 2), significant differences were noted from pre-exercise osmolality. The osmolality of LCS ingestion was significantly lower than the placebo ingestion at the Pre time point, but no significant difference was noted at the post exercise. The other electrolytes (sodium and chloride) measured in urine before and after exercise did not significantly differ between conditions. However, in the LCS ingestion, potassium measured in urine after exercise was significantly elevated compared with potassium measured in urine before exercise.

### 3.4. Effects of LCS on Oxidative Stress, Antioxidative Capacity, and Inflammation During EEE

Oxidative stress and antioxidative capacity could be assessed by TBARS, PC, GPx, SOD, and catalase (Table 3). Before exercise, no significant differences were observed in those indexes for the LCS and placebo ingestions. The level of PC in the LCS ingestion decreased slightly than placebo ingestion after EEE (*p* = 0.06). In contrast to antioxidative capacity, catalase was significantly upregulated in the LCS ingestion compared with the water placebo ingestion after EEE (*p* < 0.05, statistical power = 0.6). In addition, the LCS ingestion maintained higher antioxidant capacity than baseline levels, as measured by SOD and catalase at the Post-60 to Post-120 time points. The PC level at Post-120 also indicated lower oxidative stress than at baseline in the LCS ingestion. Table 3 shows that the inflammation marker CRP was not significantly different between treatments at all time points.

### 3.5. Effects of LCS on Aerobic Capacity

All participants completed the 70% ${\dot{V}O}_{2\max}$ exercise for 1 h, followed by 90% ${\dot{V}O}_{2\max}$ until exhaustion. Table 4 shows that no significant differences were observed in the average HR, maximal HR, running time to exhaustion, and VO_2peak_ between treatments.

## 4. Discussion

Dehydration is a critical issue during intensive and prolonged exercise training. Dehydration could affect physiological adaptation and cause health issues. The LCS in the present study was modified to modulate not only fluid replacement but also physiological antioxidation effects with effective ingestion strategies. LCS ingested 1 h before and after EEE (600 mL) could effectively attenuate fluid loss and maintain electrolyte balance. Also, exercise-associated oxidative stress could be mitigated by the upregulation of antioxidative capacity through LCS ingestion.

Water replacement is a crucial factor in reaching fluid balance for physiological homeostasis after prolonged endurance exercise. Goulet et al. [21] reviewed related studies on dehydration and endurance exercise performance in competitive athletes and recommended drinking approximately 5–10 mL/kg BW of water 2 h before exercise. The National Athletic Trainers’ Association suggested approximately 500 to 600 mL of fluid replacement 2 to 3 h before exercise for proper euhydration [22]. Individuals without knowledge of appropriate hydration strategies or who are unaware of their own fluid needs could overhydrate themselves, causing hyponatremia [23]. Furthermore, investigation of marathon runners’ hydration practices and perceptions showed that 70% of runners experienced dehydration, resulting in a major performance decrement, but only 2% reported measuring changes in BW. Validations of methods to monitor hydration status and the development of an appropriate individualized hydration strategy should be well informed [15]. Another survey study also revealed a high rate of insufficient knowledge regarding dehydration and nutrition among university and club-level athletes [24]. BW could be a useful index for degree of dehydration, and the fluid deficit during exercise was recommended to be limited to less than 2% of BW [2]. In the present study, exercising at 70% ${\dot{V}O}_{2\max}$ for 1 h followed by 90% ${\dot{V}O}_{2\max}$ until exhaustion could significantly cause fluid loss, represented by an approximate BW decrease of 2% BW from the Post-0 time point with water placebo ingestion. The dehydration status in the present study was consistent with previous recommendations, and LCS ingestion could significantly replace fluid loss compared with the placebo group during exercise (Figure 2).

Athletes often experience exercise-associated muscle cramps (EAMCs) with high-intensity or prolonged exercise because of environmental factors and as a result of muscular fatigue or lack of electrolytes such as sodium, potassium (hypokalemia), or magnesium. On the basis of physiological findings, prevention programs should be implemented to include fluid and electrolyte balance strategies and/or neuromuscular training [25]. A previous water rehydration study illustrated that muscles became more susceptible to cramping and that electrolyte solutions could reduce that effect significantly [26]. Maintaining electrolyte balance, including sodium and potassium, could be important during or after exercise to relieve cramping. Regarding nutrition, consumption of fruits such as bananas was widely applied by marathon competitors for carbohydrate and potassium supplementation to prevent cramping, but the study demonstrated that potassium was not elevated quickly enough to treat acute EAMCs, especially near the end of a competition [27]. The ORS intervention could be a potential strategy for the amelioration of electrolyte loss for physiological maintenance. Our present results show that LCS ingestion could significantly replace lost sodium and potassium and maintain their concentrations after EEE (Table 1). LCS ingestion could prevent EAMCs after exercise-associated dehydration.

Exercise-associated dehydration could also be a potential factor related to renal injury. It has been hypothesized that the mechanism is a decrease of circulatory blood volume and renal blood flow that may attenuate renal function and cause ischemic stress with temporary renal injury [28]. A study of prolonged exercise (marathon) also demonstrated a decline in eGFR and increased biomarkers for kidney injury, but these were not associated with acute exercise [29]. Nonsteroidal anti-inflammatory drugs taken by athletes for analgesic purposes may exacerbate kidney damage, especially related to severe dehydration [30]. Acute exercise could induce significant creatinine elevation and a decrease in eGFR compared with baseline levels in the present study, but these levels gradually recovered after exercise (Figure 3A,B). However, no significant difference in creatinine and eGFR was observed between the LCS and placebo groups in the present study, and the results showed that LCS ingestion did not affect renal metabolic function. In addition, CRP could be considered an inflammatory marker. A study on mountain ultramarathon participants demonstrated that CRP, creatinine, and eGFR could show early significant increases of muscle damage, inflammation, and risk for acute renal injury without any clinical symptomatology [31]. However, we found no significant CRP increment in the present study, possibly because of intensity effects (Table 3). We believe that intensity and environmental factors could be further considered for the potential benefits of LCS-related physiological effects on acute renal injury and inflammation.

Endurance exercise requires sufficient energy supply for physical maintenance, and aerobic respiration can efficiently generate energy in the mitochondria. Excessive oxygen would be necessary not only for metabolism but also for the final electron receptor as reactive oxygen species, including superoxide radical, hydroxyl radical, and hydrogen peroxide, during exercise. The moderate-intensity exercise illustrated systemic and complex health-promoting effects through the regulation and signaling of redox homeostasis [32]. The balance of oxidative stress and antioxidative capacity during high-intensity exercise is important for physiological adaptation [33]. Oxidative stress, contingent on endurance exercise duration and intensity, significantly elevates related indexes, such as oxidative markers (TBARS and protein carbonyl) and total antioxidant capacity [34]. We did not observe a significant elevation of TBARS and PC markers immediately after exercise, but the protein carbonyl marker was a slightly decrease in the LCS group than in the placebo group (*p* = 0.06) (Table 3). Regarding antioxidative capacity, catalase activity was also significantly increased in the LCS group compared with the placebo group at the Post-0 time point. In addition, SOD activity was also slightly increased in the LCS group compared with the placebo group at the Post-0 time point (*p* = 0.12). Moreover, the single EEE with LCS ingestion could also activate and maintain significant SOD activity even 2 h after exercise compared with baseline. However, previously, a limited number of reports had focused on antioxidative activities with LCS ingestion related to exercise-associated dehydration. This could have critical physiological effects on exercise performance related to electrolyte ingestion.

Different degrees of dehydration were confirmed to have deleterious effects on exercise performance. A body mass decrease of 2% during exercise could be considered an important threshold associated with impaired thermoregulatory function, elevated cardiovascular strain, and impaired aerobic exercise performance [35]. Acute and pre-exercise BW loss greater than 3% may decrease subsequent endurance performance [21], and could profoundly the performance of weight class–associated athletes. Not only aerobic capacity but also strength, power, and cognitive and motor skills were affected by dehydration [36]. In the present study, the participants ran for 1 h followed by 90% ${\dot{V}O}_{2\max}$ intensity until exhaustion. No significant differences were noted in running time until exhaustion and ${\dot{V}O}_{2\max}$ between the LCS and placebo groups (Table 4). Limitations of this study included the relatively small participant numbers, only male participants, and level of dehydration. Therefore, factors of target population, sample size, dehydration status, environment, and intensity should be further investigated for the potential effects of LCS.

## 5. Conclusions

In the present study, we found that LCS ingestion could significantly ameliorate endurance exercise–induced dehydration and maintained electrolyte homeostasis during exercise. We also employed an effective supplementation strategy (600 mL/h, 150 mL/15 min) 1 h before and after endurance exercise (>1 h). Also, the study preliminarily revealed the potential antioxidant capacity of LCS for dehydration after exercise in terms of health benefits. However, additional elements, including population, dehydration status, and environment, could be further validated for the effects of LCS ingestion.

## Figures and Tables

**Table 1:** Changes in electrolytes and osmolality after exhaustive endurance exercise.

Parameters	Baseline	Pre	Post-0	Post-60	Post-120
Na^+^ (mmol/L)					
LCS	140.25 ± 0.75 (0.54%)	140.50 ± 0.67 * (0.48%)	143.50 ± 1.83 *^,‡^ (1.28%)	141.67 ± 1.44 *^,‡,＋^ (1.01%)	141.25 ± 1.77 *^,＋^ (1.25%)
Placebo	140.50 ± 1.68 (1.19%)	140.58 ± 1.17 (0.82%)	143.92 ± 1.88 *^,‡^ (1.31%)	141.17 ± 1.27 ^‡,＋^ (0.90%)	140.75 ± 1.42 ^＋,†^ (1.01%)
Cl^−^ (mmol/L)					
LCS	102.17 ± 1.64 (1.61%)	102.92 ± 1.98 * (1.92%)	104.50 ± 1.68 *^,‡,#^ (1.61%)	103.00 ± 1.76 ^＋^ (1.71%)	102.67 ± 1.50 ^＋^ (1.46%)
Placebo	103.25 ± 1.48 (1.44%)	103.83 ± 1.40 (1.35%)	105.92 ± 1.31 *^,‡^ (1.24%)	103.42 ± 1.68 ^＋^ (1.62%)	103.33 ± 1.92 ^＋^ (1.86%)
K^+^ (mmol/L)					
LCS	3.97 ± 0.28 (6.99%)	4.07 ± 0.27 * (6.66%)	4.26 ± 0.35 * (8.28%)	4.70 ± 0.49 *^,‡,^^＋^ (10.38%)	4.50 ± 0.35 *^,‡,†^ (7.70%)
Placebo	4.08 ± 0.26 (6.34%)	4.17 ± 0.30 (7.19%)	4.30 ± 0.37 * (8.53%)	4.63 ± 0.40 *^,‡,＋^ (8.65%)	4.33 ± 0.43 ^†^ (9.91%)
O (mOsm/L)					
LCS	291.33 ± 2.10 (0.72%)	289.67 ± 2.50 * (0.86%)	299.58 ± 5.92 *^, ‡^ (1.97%)	292.92 ± 3.87 ^‡,＋^ (1.32%)	292.17 ± 4.34 ^＋^ (1.49%)
Placebo	291.92 ± 3.15 (1.08%)	290.33 ± 2.15 * (0.74%)	301.00 ± 5.36 *^,‡^ (1.78%)	291.75 ± 2.30 ^＋^ (0.79%)	290.25 ± 2.45 ^＋,†^ (0.85%)

Data are presented as mean ± standard deviation (coefficient of variation) (*n* = 12). The LCS and placebo were ingested twice: after baseline and at the Post-15 time point. The difference was considered significant when *p* was < 0.05. * Significant (*p* < 0.05) difference from the baseline. ^‡^ Significant (*p* < 0.05) difference from Pre. ^＋^ Significant (*p* < 0.05) difference from Post-0. ^†^ Significant (*p* < 0.05) difference from the Post-60. ^#^ Significant (*p* < 0.05) difference between treatments. LCS, low-osmolality carbohydrate-electrolyte solution; Na^+^, sodium; Cl^−^, chlorine; K^+^, potassium; O, osmolality; Pre, pre-exercise; Post-0, immediately after exercise; Post-60, 60 min after exercise; Post-120, 120 min after exercise.

**Table 2:** Changes in electrolytes and osmolality of urine after exhaustive endurance exercise.

Parameters	Pre-U	Post-U
Na^+^ (mmol/L)		
LCS	41.71 ± 14.95 (35.84%)	62.00 ± 47.22 (76.17%)
Placebo	56.86 ± 24.35 (42.83%)	60.29 ± 43.64 (72.39%)
Cl^−^ (mmol/L)		
LCS	43.00 ± 12.94 (30.08%)	63.43 ± 46.60 (73.46%)
Placebo	61.43 ± 30.03 (48.89%)	59.14 ± 37.03 (62.61%)
K^−^ (mmol/L)		
LCS	10.54 ± 6.31 (59.83%)	33.33 ± 13.96 * (41.87%)
Placebo	15.60 ± 7.12 (45.63%)	28.79 ± 16.01 (55.61%)
O (mOsm/L)		
LCS	294.43 ± 141.49 ^#^ (48.06%)	350.10 ± 212.31 (60.64%)
Placebo	349.43 ± 127.07 (36.36%)	361.00 ± 230.49 (63.85)

The data are presented mean ± standard deviation (coefficient of variation) (*n* = 7). Pre-U and Post-U represent the urine collection period during LCS and placebo ingestion before and after exhaustive endurance exercise. * Significant (*p* < 0.05) difference from Pre-U. ^#^ Significant (*p* < 0.05) difference between conditions. LCS, low-osmolality carbohydrate–electrolyte solution; Na^+^, sodium; Cl^−^, chlorine; K^+^, potassium; O, osmolality; Pre-U, pre-exercise urine measurement; Post-U, post-exercise urine measurement.

**Table 3:** Changes in oxidative stress, antioxidative capacity, and inflammation after exhaustive endurance exercise.

Parameters	Baseline	Pre	Post-0	Post-60	Post-120
TBARS (µM)					
LCS	2.73 ± 0.52 (18.97%)	2.79 ± 0.50 (17.89%)	2.95 ± 0.70 (23.81%)	2.68 ± 0.57 (21.40%)	2.89 ± 0.54 (18.74%)
Placebo	2.73 ± 0.44 (16.01%)	2.85 ± 0.61 (21.46%)	2.93 ± 0.49 (16.66%)	2.88 ± 0.66 (22.97%)	2.97 ± 0.60 (20.23%)
PC (nmol carbonyl/mg protein)					
LCS	3.02 ± 0.58 (19.11%)	2.87 ± 0.37 (13.05%)	2.72 ± 0.31 *^,#^ (11.52%)	2.89 ± 0.52 (18.12%)	2.77 ± 0.31 * (11.04%)
Placebo	3.08 ± 0.67 (21.83%)	3.01 ± 0.50 (16.54%)	2.89 ± 0.43 (14.80%)	2.89 ± 0.46 (15.93%)	3.03 ± 0.98 (32.30%)
GPx (U)					
LCS	1076.30 ± 180.54 (16.77%)	1249.87 ± 195.95 (15.68%)	1239.71 ± 275.29 (22.21%)	1226.17 ± 256.26 (20.90%)	1289.97 ± 214.49 (16.63%)
Placebo	1270.44 ± 237.65 (18.71%)	1313.28 ± 295.39 (22.49%)	1364.84 ± 301.27 (22.07%)	1347.40 ± 251.12 (18.64%)	1274.61 ± 290.00 (22.75%)
SOD (U/mL)					
LCS	5.18 ± 0.80 (15.45%)	5.43 ± 0.56 (10.27%)	6.15 ± 0.89 *^,‡^ (14.49%)	5.94 ± 0.89 *^,‡^ (14.93%)	6.02 ± 1.00 *^,‡^ (16.63%)
Placebo	5.34 ± 0.51 (9.47%)	5.40 ± 0.63 (11.65%)	5.78 ± 0.78 (13.47%)	5.95 ± 1.30 (21.82%)	5.60 ± 0.56 (9.96%)
CAT (nmol/min/mL)					
LCS	1270.56 ± 246.95 (19.44%)	1340.99 ± 266.67 (19.89%)	2046.21 ± 381.98 *^,‡,#^ (18.67%)	1565.47 ± 367.23 *^,‡,＋^ (23.46%)	1677.25 ± 395.63 *^,‡,＋^ (23.59%)
Placebo	1337.18 ± 386.32 (28.89%)	1471.97 ± 302.45 (20.55%)	1820.37 ± 417.35 *^,‡^ (22.93%)	1623.01 ± 443.46 (27.32%)	1694.80 ± 419.63 *^,‡^ (24.76%)
CRP (mg/dL)					
LCS	0.06 ± 0.06 (95.43%)	0.06 ± 0.06 (97.85%)	0.07 ± 0.06 *^,‡^ (96.28%)	0.06 ± 0.06 ^＋^ (94.18%)	0.06 ± 0.06 ^＋^ (96.68%)
Placebo	0.08 ± 0.13 (165.62%)	0.08 ± 0.13 (163.30%)	0.09 ± 0.14 (159.70%)	0.08 ± 0.13 (163.03%)	0.08 ± 0.14 (162.88%)

The data are presented as mean ± standard deviation (coefficient of variation) (*n* = 12). LCS and placebo were ingested twice: after baseline and immediately after exercise. The difference was considered significant when *p* was < 0.05. * Significant (*p* < 0.05) difference from baseline. ^‡^ Significant (*p* < 0.05) difference from Pre. ^＋^ Significant (*p* < 0.05) difference from Post-0. ^#^ Significant (*p* < 0.05) difference between treatments. LCS, low-osmolality carbohydrate–electrolyte solution; TBARS, thiobarbituric acid reactive substances; PC, protein carbonyl; GPx, glutathione peroxidase; SOD, superoxide dismutase; CAT, catalase; CRP, C-reactive protein; Pre, pre-exercise; Post-0, Immediately after exercise; Post-60, 60 min after exercise; Post-120, 120 min after exercise.

**Table 4:** Changes in aerobic capacity after exhaustive endurance exercise.

Parameters	Exhaustion (min)	MHR (beats/min)	${\dot{V}O}_{2\mathrm{peak}}$ (mL/kg/min)
LCS	63.13 ± 3.35 (5.30%)	198.58 ± 9.97 (5.02%)	52.33 ± 6.66 (12.72%)
Placebo	63.28 ± 3.37 (5.33%)	198.00 ± 9.49 (4.79%)	53.11 ± 6.44 (12.13%)

The data are presented as mean ± standard deviation (coefficient of variation) (*n* = 12). Time until exhaustion was recorded for running at 90% ${\dot{V}O}_{2\max}$ immediately after 60 min of exercise at 70% ${\dot{V}O}_{2\max}$. LCS, low-osmolality carbohydrate–electrolyte solution; MHR, maximal heart rate; ${\dot{V}O}_{2\mathrm{peak}}$, peak oxygen intake.
